# Supplementary material for: Associations of Diabetes and Hyperglycaemia with Extent and Outcomes of Acute Burn Injuries
Source: Biomedicines. 2024 May 19;12(5):1127. doi: 10.3390/biomedicines12051127 (PMC11118006; doi:10.3390/biomedicines12051127)
Supplement: Supplementary file 1 [file biomedicines-12-01127-s001.zip › biomedicines-2969429-supplementary.pdf]

Supplementary Material

"Associations of diabetes and hyperglycaemia with extent and outcomes of acute burn injuries" by

Jeffrey Chandra, Edward Raby, Fiona M. Wood, P. Gerry Fegan & Bu B. Yeap

Results

Baseline diabetes therapy

3 patients had Type 1 diabetes: 2 receiving a basal-bolus regimen, the other using an insulin pump. In 26 patients diagnosed with Type 2 diabetes, 8 were on monotherapy, 10 dual therapy, 5 triple and 1 quadruple therapy; these included 11 patients receiving insulin. Two were diet-controlled.

Initiation of insulin therapy

Two patients with known diabetes who were insulin naive required insulin treatment, being given on average 4 and 11 IU/day. Five patients with no history of diabetes who had BSL monitoring received insulin during their stay, being given 14, 15, 16, 35 and 122 IU/day.

Treatment of patients with known diabetes

Six patients with a history of diabetes did not experience any hyperglycaemia. Their existing diabetes treatment regimens included 3 on metformin monotherapy, and one each on metformin and one or two additional oral agents or insulin. In the 24 patients with a history of diabetes who did experience hyperglycaemia, 18 were admitted on oral hypoglycaemic agents. Of these, 9 patients had insulin included in their regimen, 4 patients had metformin monotherapy, 3 received metformin and an additional agent while 2 received metformin with 2 additional agents.

Table S1. Patients were categorised according to diabetes status based on history of diabetes and admission HbA1c, and whether they had inpatient BSL monitoring. Data are shown as median (25th and 75th percentiles), mean (SD) or percentage (N).

	**Undiagnosed diabetes (no history of diabetes, admission HbA1c ≥6.5%)**		**Pre-existing diabetes (history of diabetes OR admission HbA1c ≥6.5%)**		**No diabetes (no history of diabetes and admission HbA1c <6.5%)**	
Inpatient BSL monitoring	**Yes**	**No**	**Yes**	**No**	**Yes**	**No**
N	3 (M=3 ,F=0)	1 (M=1, F=0)	32 (M=24, F=8)	2 (M=2, F=0)	18 (M=12, F=6)	99 (M=69, F=30)
Age (years)	51	39	55	53	52	43
TBSA (%)	8.0 (5.3, 8.8)	4.0	1.0 (0.5, 3.0)	4.0 (4.0, 4.0)	18 (12, 27)	2.5 (1.0, 4.0)
% patients on NGT nutrition	33% (1)	0	6.3% (2)	0	61% (11)	0
LOS (days)	10 (8.0, 11)	5	6 (4.0, 10)	5 (5.0, 5.0)	19 (15, 40)	5 (3.0, 8.0)
Admission HbA1c (%)	7.7 (7.2, 10)	7.1	8.4 (7.1, 9.6)	6.1 (5.6, 6.6)	5.4 (5.3, 5.8)	5.2 (5.0, 5.6)
BSL measurements	105	0	784	0	434	0
Mean BSL (mmol/L)	13 (5.5)	N/A	10 (4.1)	N/A	8.7 (2.9)	N/A
BSL ≥11.1 mmol/L	59% (62)	0	31% (246)	0	14% (59)	0
BSL ≥16 mmol/L	24% (25)	0	8.7% (68)	0	3.0% (13)	0
BSL ≤4 mmol/L	1.0% (1)	0	2.3% (18)	0	0	0
Median days with BSL monitoring	10 (4,11)	0	6 (3.8, 10)	0	8 (2.8,13)	0
Days with BSL measurement	25	0	226	0	138	0
Days with BSL ≥11.1 mmol/L	80% (20)	0	52% (117)	0	20% (27)	0

Table S2. Patients with known diabetes or undiagnosed diabetes (admission HbA1c ≥6.5%) (A) and without a history of diabetes with an admission HbA1c <6.5% (B) who received inpatient BSL monitoring, stratified according to %TBSA. Data are shown as percentage (%) and number (n) of BSL events, and days with BSL ⩾11.1 mmol/L.

A

**TBSA**	**N**	**Total BSLs**	**% of BSL events (N)**			**Total days with BSLs**	**Days with BSL ≥11.1 mmol/L**
			**≥11.1 mmol/L**	**≥16 mmol/L**	**≤4 mmol/L**		
**<1%**	14	307	30% (92)	6.5% (20)	1.6 (5)	85	51% (43)
**1 to <2%**	5	150	27% (40)	8.7% (13)	0% (0)	47	43% (20)
**2 to <5%**	5	94	18% (17)	5.3% (5)	1.1% (1)	30	40% (12)
**5 to 10%**	4	179	45% (80)	15% (26)	6% (11)	45	73% (33)
**≥10%**	3	46	28% (13)	8.7% (4)	2.3% (1)	16	44% (7)
**Unknown**	1	8	50% (4)	0	0	3	25% (2)
**Total**	**32**	**784**	**31%** (246)	**8.7%** (68)	**2.3% (18)**	**226**	**52%** (117)

B

**<1%**	0	0	0% (0)	0% (0)	0% (0)	0	0% (0)
**1 to <2%**	0	0	0% (0)	0% (0)	0% (0)	0	0% (0)
**2 to <5%**	2	19	16% (3)	0% (0)	0% (0)	6	33% (2)
**5 to <10%**	2	12	0% (0)	0% (0)	0% (0)	5	0% (0)
**≥10%**	14	403	14% (56)	3.2% (13)	0% (0)	127	20% (25)
**Unknown**	0	0	0% (0)	0% (0)	0% (0)	0	0% (0)
**Total**	**18**	**434**	**14%** (59)	**3.0%** (13)	**0% (0)**	**138**	**20%** (27)

Table S3. Associations of variables with risk of length of stay (LOS) during hospitalization for acute burn injury, in patients with HbA1c (N=149). Data are shown as regression coefficients for change in outcome counts using Negative Binomial regression, with 95% confidence intervals, and p-values for individual factors.

**Variables***	**Negative Binomial** [a]
**Intercept**	4.09 (3.09, 5.40)
**Diabetes/monitoring status**	
No history of diabetes, BSL not monitored	Reference
No history of diabetes, BSL monitored	1.55 (1.08, 2.23), p = 0.027
Known diabetes, BSL monitored	
**Inpatient hyperglycaemia**	
Inpatient hyperglycaemia	1.28 (1.01, 1.63), p = 0.037
**Nutrition Route**	
Oral	
NGT	2.20 (1.38, 3.53), p = 0.002
**TBSA**	
TBSA	1.02 (1.00, 1.04), p = 0.010
**Age**	
Age	1.01 (1.00, 1.01), p = 0.007
**Sex**	
Male	
Female	

* Backward selection was performed to create further models. Selection ceased when AIC was not improved with further elimination.
a Multivariate Negative Binomial Regression model

Table S4. Patients were categorised according to diabetes status based on history of diabetes and admission HbA1c, and whether they had inpatient BSL monitoring. Numbers of patients who experienced any BSL ≥11.1 mmol/L, no BSL ≥11.1 mmol/L or who had no BSL measurement, are tabulated.

	**Undiagnosed diabetes (no history of diabetes, admission HbA1c ≥6.5%)**	**Pre-existing diabetes (history of diabetes OR admission HbA1c ≥6.5%)**	**No diabetes (no history of diabetes and admission HbA1c <6.5%)**
Any BSL ≥11.1 mmol/L	3	24	8
No BSL ≥11.1 mmol/L	0	5	10
No BSL measurement	1	1	99
